# Optimization of Fermentation Process of Pomegranate Peel and *Schisandra Chinensis* and the Biological Activities of Fermentation Broth: Antioxidant Activity and Protective Effect Against H_2_O_2_-induced Oxidative Damage in HaCaT Cells

**DOI:** 10.3390/molecules26113432

**Published:** 2021-06-05

**Authors:** Hui-Min Liu, Peng-Fei Xu, Ming-Yan Cheng, Sheng-Nan Lei, Qing-Lei Liu, Wei Wang

**Affiliations:** 1 School of Perfume & Aroma and Cosmetics, Shanghai Institute of Technology, Shanghai 201418, China; szliuhm@sit.edu.cn (H.-M.L.); 186071227@mail.sit.edu.cn (P.-F.X.); 206071241@mail.sit.edu.cn (M.-Y.C.); 196071222@mail.sit.edu.cn (S.-N.L.); liuqinglei@sit.edu.cn (Q.-L.L.); 2 Engineering Research Center of Perfume & Aroma and Cosmetics, Ministry of Education, Shanghai 201418, China

**Keywords:** pomegranate peel, *Schisandra Chinensis*, fermentation, response surface method, antioxidant activity, ROS

## Abstract

In this study, the lactobacillus fermentation process of pomegranate (*Punica granatum* L.) peel and *Schisandra chinensis* (Turcz.) Baill (PP&SC) was optimized by using the response surface method (RSM) coupled with a Box-Behnken design. The optimum fermentation condition with the maximal yield of ellagic acid (99.49 ± 0.47 mg/g) was as follows: 1:1 (*w:w*) ratio of pomegranate peel to *Schisandra chinensis*, 1% (*v:v*) of strains with a 1:1 (*v:v*) ratio of *Lactobacillus Plantarum* to *Streptococcus Thermophilus*, a 37 °C fermentation temperature, 33 h of fermentation time, 1:20 (g:mL) of a solid–liquid ratio and 3 g/100 mL of a glucose dosage. Under these conditions, the achieved fermentation broth (FB) showed stronger free radical scavenging abilities than the water extract (WE) against the ABTS^+^, DPPH, OH^−^ and O_2_^−^ radicals. The cytotoxicity and the protective effect of FB on the intracellular ROS level in HaCaT cells were further detected by the Cell Counting Kit-8 (CCK-8) assay. The results showed that FB had no significant cytotoxicity toward HaCaT cells when its content was no more than 8 mg/mL. The FB with a concentration of 8 mg/mL had a good protective effect against oxidative damage, which can effectively reduce the ROS level to 125.94% ± 13.46% (*p* < 0.001) compared with 294.49% ± 11.54% of the control group in H_2_O_2_-damaged HaCaT cells. The outstanding antioxidant ability and protective effect against H_2_O_2_-induced oxidative damage in HaCaT cells promote the potential for the FB of PP&SC as a functional raw material of cosmetics.

## 1. Introduction

Pomegranate is an ancient fruit that has been studied by many researchers [1], and its seeds, leaves, peel and flowers can be used as herbal medicine with the functions of arresting thirst, astringency, diarrhea and hemostasis [2]. Pomegranate is rich in a variety of antioxidant substances with the effects of scavenging free radicals [3], antiaging [4] and whitening [5]. Pomegranate extracts from seeds and peels have been used in many industries, such as foods [6], medicine [7] and cosmetics [8], due to their good characteristics and development of deep processing technology. Ellagic acid is one of active substances [9] of pomegranates. Besides its good activity of antioxidation and cells protection [10], ellagic acid may be a potential drug to limit the burden of diabetes [11].

*Schisandra chinensis* is a dry and mature fruit of perennial deciduous woody vines [12], and it has been used as a Chinese traditional herbal medicine [13]. *Schisandra chinensis* is rich in lignans, which have a variety of pharmacological effects and display good therapeutic effects on many diseases [14]. The research on *Schisandra chinensis* has mainly been concentrated on its composition analysis and biological activity. Studies have proven that *Schisandra chinensis* has the effects of sedation [15], anti-inflammatory [16], inhibiting gene expression [17] and protecting cells [18].

Fermentation is a physiological activity of microorganisms [19], and microbial fermentation technology has been widely used in foods [20], pharmaceuticals [21], energy [22], agriculture [23] and environmental protection [24]. In recent years, there have been some research on pomegranates as a fermentation substrate, mostly in food [25], health products [26] and so on. However, there are few studies on the fermentation of *Schisandra chinensis* and even less research about the combination of pomegranates and *Schisandra chinensis*. A lot of research has proven that enzymes can degrade the fibrous tissue of plants, resulting in cell rupture and the outflow of intracellular nutrients in the process of fermentation [27]. Therefore, the extraction rate of effective components and its efficacy may be improved [28]. Macromolecular substances in plants cannot be easily absorbed by the skin, and these substances can be degraded into small molecules easily absorbed by the skin through microbial fermentation, which can also increase the utilization rate of the effective ingredients [29]. Due to the rich active components of plants, such as natural flavonoids, polysaccharides, proteins, polyphenols and new metabolites of microorganisms retained in FB, the research on cosmetics using FB as functional raw materials has attracted great attention. It was reported that FB produced by probiotic fermentation are more friendly and safer to the human skin [30]. Fermentation technology has a unique advantage in plant extraction.

The response surface method (RSM) develops in 50 s, and it is widely used in many technical fields [31], especially in process design and optimization [32]. RSM provides optimization with the help of polynomials adapted to the data obtained from optimization procedure-designed experiments. In this study, the RSM was used to evaluate the effects of different variables on the outcome, which is the ellagic acid content and the interactions among different variables. Oxidative stress was caused by the disruption of the balance between oxidative and antioxidant systems [33]. Excessive reactive oxygen species (ROS) may lead to the oxidation of polyunsaturated fatty acids in low-density lipoprotein or cell membrane phospholipids [34]. This kind of lipid peroxidation produces a large number of peroxidation products that may destroy the structural integrity of the cell membrane and eventually lead to oxidative damage [35]. The level of ROS in cells can be used as an indicator of the protective ability of the active substance [36]. More and more studies have been carried out about how the active substance reduces the UVA/UVB or H_2_O_2_-induced oxidative damage in human cells [37].

Hence, the focus of this paper aims to: (1) develop and optimize the fermentation conditions of pomegranate peels and *Schisandra chinensis* (PP&SC) by using response surface methodology with a Box-Behnken design, (2) quantifying the ellagic acid in the FB and WE by HPLC and determining the scavenging ability of the various free radicals and (3) evaluating the cytotoxicity and the protective effect of FB and WE on the intracellular ROS level in HaCaT cells.

## 2. Results and Discussion

### 2.1. Single-Factor Experiments

**Figure 1:**
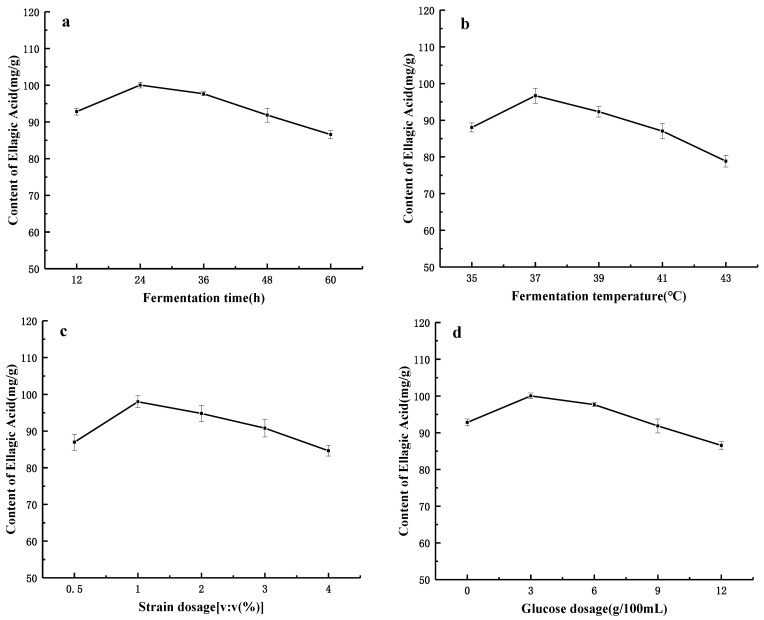

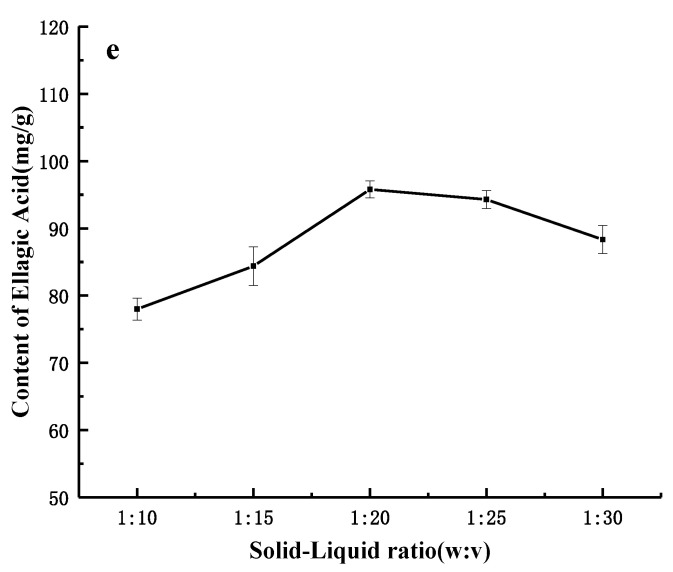
The influence of each single factor on the content of ellagic Acid in FB. (**a**) Fermentation time (h), (**b**) fermentation temperature (°C), (**c**) strain dosage (*v:v*), (**d**) glucose dosage (g/100 mL) and (**e**) solid–liquid ratio (*w:v*). 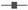 Content of ellagic acid. Data shown as the mean ± S.D. (*n* = 5).

Single-factor experiments and RSM were applied to the optimization of fermentation. Single-factor experiments were carried out with the selected parameters and levels, which provided a suitable range for the BBD in the RSM. To explore the influence of different conditions on the FB, we developed five factors and five levels of experimental program in the single-factor experiments. The ellagic acid content, lignans content and the total DPPH clearance rate were detected in all single-factor experiments. As shown in Figure 1a, the ellagic acid content first increased with the fermentation time from 12 h to 36 h, then fell when the fermentation time was in the range of 36–60 h. A similar change in the trend of the ellagic acid content appeared for the influences of fermentation temperature, strain dosage, glucose dosage and the solid–liquid ratio (Figure 1b–e). The ellagic acid content reached the maximum when the glucose dosage, fermentation time and fermentation temperature reached 3 g/100 mL, 36 h and 37 °C, respectively. A suitable glucose additional amount, fermentation time and fermentation temperature can benefit the growth and activity of microorganisms, thus affecting the release and hydrolysis of tannin into ellagic acid. However, excessive fermentation time and fermentation temperature partly may be attributed to the loss of ellagic acid due to its instability, such as oxidation or degradation to form other compounds. Glucose can be used as the main carbon source of *Lactobacillus plantarum* and *Streptococcus thermophilus*, but an excessive glucose dosage can affect the utilization of fermentation substrates by microorganisms, resulting in the reduced release of active substances from plant tissue. In the case of Tunisia soft-seed pomegranates, the contents of ellagic acid in the ethanol, aqueous and ethyl acetate extracts of the pomegranate peels were reported to be 28.45, 41.36 and 32.68 mg/g, respectively [38]. As a contrast, in this experiment, the ellagic acid content was significantly improved after fermentation.

The experimental design of the process optimization by using RSM with BBD was based on the results of the single-factor tests. In this experiment, we mainly explored the change of the ellagic acid content in the FB and the antioxidant capacity of the FB in different fermentation conditions. Therefore, we chose the following three factors that had a greater impact on the ellagic acid content as the response surface variables: fermentation temperature, fermentation time and glucose dosage.

### 2.2. Building Models and Analyzing Statistics

Based on the results of the experiments on a single factor, three parameters: glucose dosage (A), fermentation time (B) and fermentation temperature (C) were selected as the variables for optimizing the process of fermentation using the ellagic acid content as the index. The experimental steps and their corresponding experimental results are shown in Table 1.

According to the test method designed by design-expert 7.0, the fitting regression of several data in Table 1 was performed. As in Table 2, a multiple regression analysis was used to analyze the experimental data, and the relationship between the response variables and the quadratic regression equation was obtained (Equation (1)).

Y(content of ellagic acid) = 96.43 + 1.20A − 1.97B + 0.86C − 0.92AB − 0.70AC − 0.24BC − 5.23A^2^ − 4.98B^2^ − 8.38C^2^ (1)

The significance of the model was detected, and the results in Table 2 were obtained. The high correlation coefficient value (R^2^ = 0.982) showed that the experimental response values have a good correlation with the predicted response values. *p* < 0.001 indicated that each dependent variable had a significant relationship with all the independent variables, and the established model was meaningful. The missing item, *p* > 0.05, indicated that the model fit well and had no significant differences, so the content of ellagic acid could be analyzed by using the model and regression equation. According to the *p*-value, we determined that three linear coefficients (A, B and C); three quadratic coefficients (A^2^, B^2^ and C^2^) and three interaction coefficients (AB, AC and BC) were significant, which indicated that there was an interaction between the tested variables. The F-value of the model was 50.22, which indicated that the model was very significant when *p* < 0.0001. Due to the relative pure error, the lack of fitting F-value of 4.89 was not significant (*p* < 0.37951). The suitability of the models must be checked to prevent inappropriate models from producing undesirable or misleading results [39].

**Figure 2:**
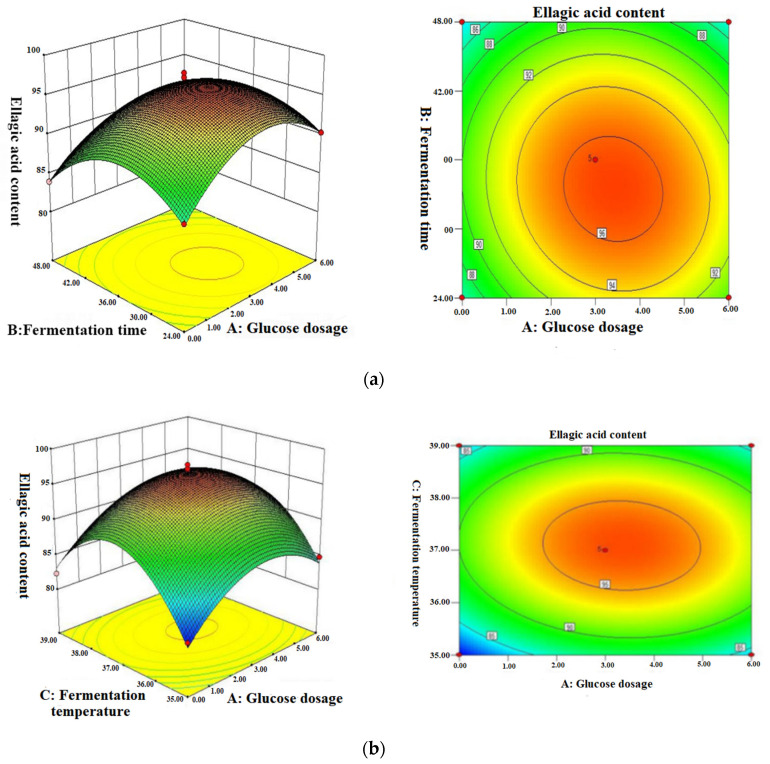

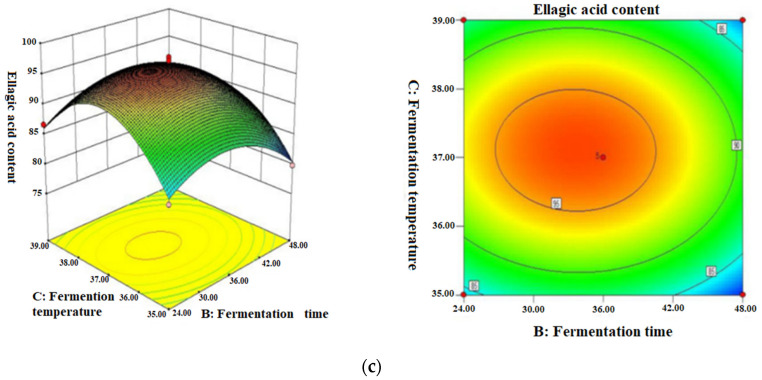
Response surface plots: (**a**) glucose dosage and fermentation time on the ellagic acid content, (**b**) glucose dosage and fermentation temperature on the ellagic acid content and (**c**) fermentation time and fermentation temperature on the ellagic acid content.

According to the results of the regression analysis, the response surface contour map and the response surface 3D map were drawn in Figure 2. The three-dimensional diagram intuitively reflected the influence of the interaction of each factor on the response value and found the best parameters and the interactions among the parameters. Figure 2a shows the effects of the fermentation time and glucose dosage on the ellagic acid content. The content of ellagic acid increased with the extension of the fermentation time. When the amount of glucose reached a certain point, the content of ellagic acid decreased with the extension of the fermentation time or the increase of glucose. Similarly, the change of the ellagic acid content showed the same trend in Figure 2b,c. The best process conditions we got through optimization were as follows: 6 g/100 mL of glucose addition, 33 h of fermentation time and 37 °C of fermentation temperature. Milessi et al.’s research proved that adding suitable carbon sources can make microbes proliferate, but excessive carbon sources will reduce the utilization rate of microorganisms to fermentation substrates [40]. Lu et al. found that a proper fermentation time can enable microorganisms to proliferate and make full use of fermentation substrates [41]. The temperature suitable for microbial growth is crucial [42]. High or low temperatures may lead to the activity of microorganisms being reduced.

According to the response surface experiment, the optimal technological conditions for the fermentation of PP&SC were as follows: the glucose dosage was 6 g/100 mL, the fermentation time 33 h, the fermentation temperature 37 °C, the strained content (*v:v*) 1% and solid–liquid ratio 1:20 (g:mL). Under these conditions, the contents of ellagic acid and lignans in the FB reached 99.49 ± 0.47 mg/g and 60.03 ± 0.69 mg/g, respectively, with a DPPH scavenging rate of 95.70% ± 0.36% at the diluted concentration of 0.9 mg/mL. The experiment was repeated three times, and the average value was taken to verify the good effects of the experiment.

### 2.3. HPLC Analysis of Ellagic Acid in the FB and WE

**Figure 3:**
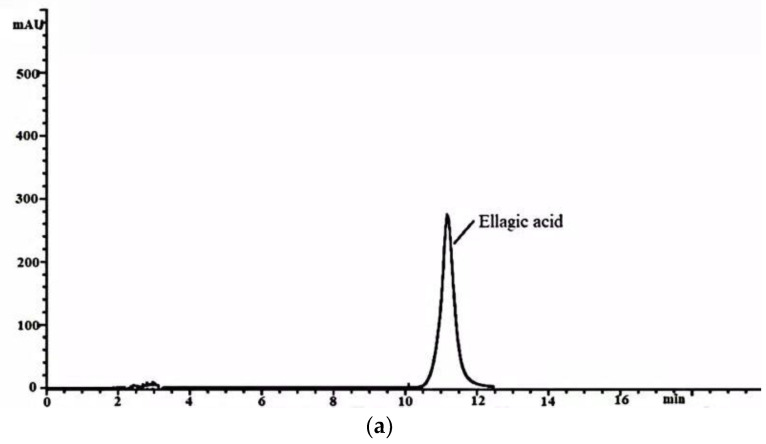

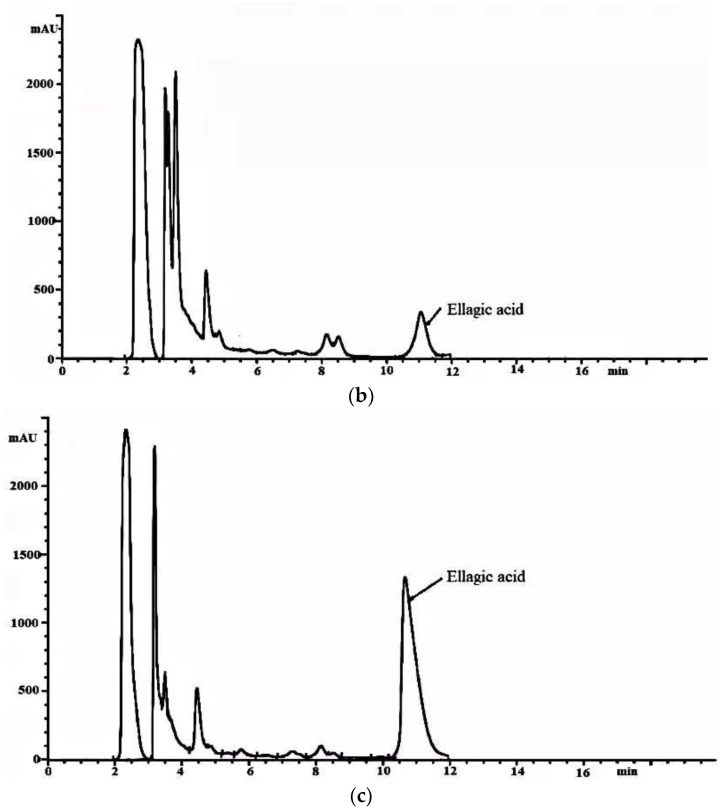
HPLC chromatograms of ellagic acid. (**a**) Ellagic acid standard, (**b**) ellagic acid in WE and (**c**) ellagic acid in FB.

The presence of ellagic acid in the FB and WE was detected by HPLC analysis, according to the retention time of the ellagic acid samples (Figure 3). The ellagic acid present in the FB and WE exhibited the same retention time as that of the standard ellagic acid. The results showed that the content of ellagic acid in the FB significantly increased after fermentation compared to that in the WE. The content of ellagic acid was 34.88 ± 0.76 mg/mL in the WE and 99.49 ± 0.47 mg/mL in the FB. These results were consistent with previous studies that microbial fermentation may reduce the pH of FB and promote the conversion of tannin acid to ellagic acid [43].

### 2.4. Antioxidant Ability of WE and FB

**Figure 4:**
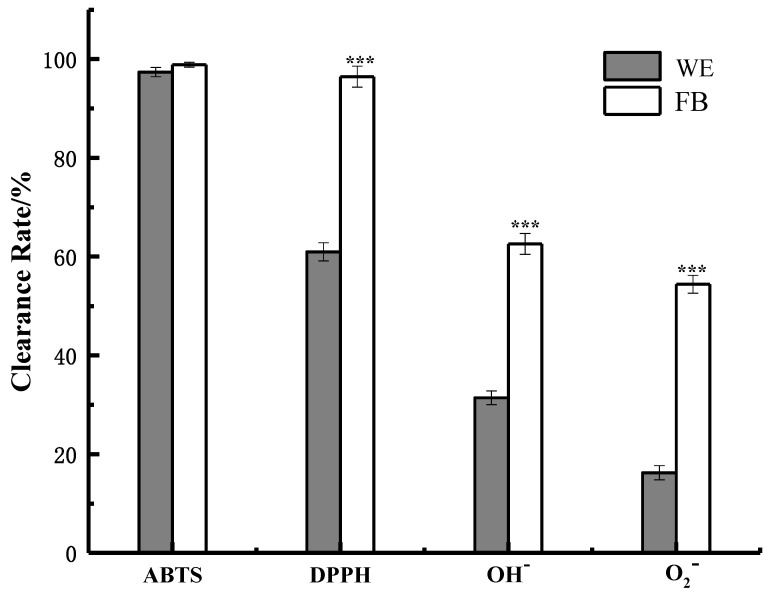
Antioxidant ability of the WE and FB. Significance: *** *p* < 0.001 vs. WE-treated group. Data shown as the mean ± S.D. (*n* = 3).

To explore in vitro antioxidant activity of the FB, the free radical scavenging abilities against the ABTS^+^, DPPH, OH^−^ and O_2_^−^ radicals were detected compared with those of WE. The results are shown in Figure 4. When the FB was diluted to 0.6 mg/mL, 0.9 mg/mL, 0.05 g/mL and 0.66 g/mL, the FB still showed strong scavenging abilities against the ABTS, DPPH, OH^−^ and O_2_^−^ radicals, with scavenging rates of 93.00% ± 0.56%, 95.0% ± 0.56% (*p* < 0.001), 79.50% ± 0.78% (*p* < 0.001) and 70.80% ± 0.32% (*p* < 0.001), respectively. Particularly, the EC_50_ value of the DPPH scavenge activity of freeze-dried FB was 0.042 ± 0.001 mg/mL, which was a little lower than that of pure Vitamin C (EC_50_ = 0.050 mg/mL). In addition, the EC_50_ value of the ABTS, OH^−^ and O_2_^−^ scavenging activity of freeze-dried FB were as follows: 0.033 ± 0.001 mg/mL, 4.481 ± 0.032 mg/mL and 6.002 ± 0.040 mg/mL. We also found that the content of ellagic acid effectively increased with the presence of both pomegranate peels and *Schisandra chinensis* (1:1 *w:w*) compared with the situation with the presence of a pure pomegranate peel. Under the same concentration of the WE and FB, the latter showed stronger free radical scavenging abilities. Microbial fermentation is a complex process, during which microorganisms may decompose, transform or synthesize different substances through their own metabolic activities [44]. In this case, microbial fermentation can enhance the antioxidant activities through various ways. It may promote cell rupture and the outflow of intracellular antioxidant substances in the plant, such as ellagic acid, lignans, flavonoids and so on. It may also promote the conversion of tannin acid to ellagic acid, resulting in an increase in the content of ellagic acid. On the other hand, microbial fermentation may produce antioxidant factors in its biochemical process [45].

### 2.5. Effects of FB, WE and H_2_O_2_ on the Activity of HaCaT Cells

**Figure 5:**
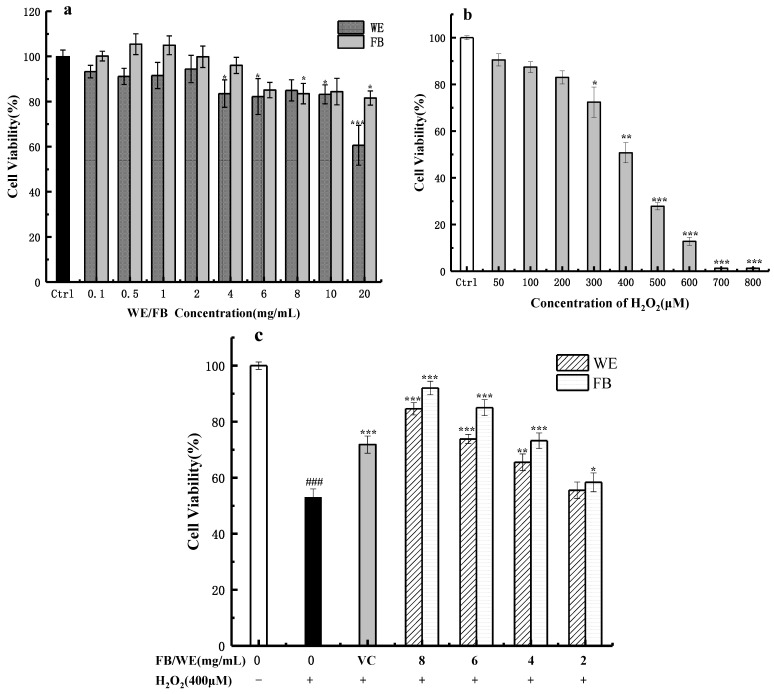
Effects of WE and FB on H_2_O_2_-introduced HaCaT cells damage. (**a**) Cytotoxicity of WE and FB on HaCaT cells. The HaCaT cells were cultured for 24 h with the WE and FB of 20, 10, 8, 6, 4, 2, 1, 0.5 and 0.1 mg/mL, and then, the cell viability was measured by a CCK-8 assay. Significance: *** *p* < 0.001 and * *p* < 0.05 vs. the control group. (**b**) Cytotoxicity of different concentrations of H_2_O_2_ on HaCaT cells. The HaCaT cells were cultured in complete medium for 24 h and then stimulated with different concentrations (50, 100, 200, 300, 400, 500, 600, 700 and 800 μM) of H_2_O_2_ for 2 h, and then, the cell viability was measured by the CCK-8 assay. Significance: *** *p* < 0.001, ** *p* < 0.01 and * *p* < 0.05 vs. the control group. (**c**) Cytotoxicity of the WE and FB on 400 μM H_2_O_2_-introduced HaCaT cell damage. The cells were cultured with different concentrations (8, 6, 4 and 2 mg/mL) of WE and FB for 24 h and then stimulated by 400 μM H_2_O_2_ for 2 h, and then, the cell viability was measured by the CCK-8 assay. In the picture: VC stands for Vitamin C, and the concentration is 2 μg/mL. Significance: ^###^ *p* < 0.001 vs. the control group. *** *p* < 0.001, ** *p* < 0.01 and * *p* < 0.05 vs. the H_2_O_2_-treated group. Data shown as the mean ± S.D. (*n* = 5).

The CCK-8 assay was used to determine the cytotoxicity of the WE, FB and H_2_O_2_ on the HaCaT cells. As shown in Figure 5a, the viability of the HaCaT cells gradually decreased in the groups treated with FB and WE at concentrations of 0.1~20 mg/mL. When the concentrations of WE and FB were in the range of 0.1~10 mg/mL, the corresponding cell viability was higher than 80% of the control group within 24 h. Furthermore, in the concentration range of 0.1~8 mg/mL, the cell viability for the FB was higher than 90% of the control group, indicating no significant cytotoxicity. In particular, when the concentration of the FB was in the lower range of 0.1~2 mg/mL, the cell viability for the FB was above 100% compared with the control group, indicating FB can promote the growth of HaCaT cells in low concentrations. The higher the dose of WE or FB, the more cytotoxicity was observed, since the cell viability decreased to 60.64% ± 8.83% (*p* < 0.001) and 81.56% ± 3.15% (*p* < 0.05) following the exposure to 20 mg/mL of WE and FB, respectively. We obviously found that, at the same concentration, the cell viability of the FB was lower than that of the WE, indicating that FB is milder to the HaCaT cells. It was reported that Lactobacillus fermentation has the ability to reduce the cytotoxicity of the fermentation substrates [46]. Microorganisms can decompose toxic substances or modify their toxic components to reduce or eliminate their toxicity [47]. A variety of plant extracts and FB were reported to promote the growth of cells [48] due to the rich bioactive substances, with small molecule produced in the process of plant growth and development, such as flavonoids, tannins and alkaloids [49].

To confirm a suitable concentration for the H_2_O_2_-induced injury model in vitro, nominal concentrations of H_2_O_2_ (50–800 μM) exposed to HaCaT cells were measured by the CCK-8 assay [50]. As shown in Figure 5b, the viability of the HaCaT cells was dose-dependently decreased and the concentration of 400 μM H_2_O_2_ caused about 50% cell mortality. As Zhang et al. [50] reported, at the concentration of 50% cell mortality, the ROS level was high, and the cells received appropriate damage. Therefore, 400 μM H_2_O_2_ was selected as the optimal cell injury concentration for all the subsequent experiments.

The results showed that, before the H_2_O_2_ treatment, the HaCaT cells were cultured in 8, 6, 4 and 2 mg/mL of WE and FB and then treated with 400 μM of H_2_O_2_ [50]. As shown in Figure 5c, the pretreatment of HaCaT cells with FB and WE in the range of 2~8 mg/mL increased the cell viability in different degrees compared with the control group. Among them, the cell viabilities of the FB pretreatment groups (2, 4, 6 and 8 mg/mL) significantly increased from 52.97% ± 3.06% in the injury group to 58.36% ± 3.37% (*p* < 0.05), 73.22% ± 2.79% (*p* < 0.001), 85.01% ± 2.85% (*p* < 0.001) and 92.03% ± 2.42% (*p* < 0.001), respectively. The FB pretreatment at 6 or 8 mg/mL yielded superior protective effects in contrast with 2 μg/mL of the VC treatment group (71.82% ± 1.60%, *p* < 0.001). Hseu et al. [51] reported that the ellagic acid treatment significantly inhibited the UVA-induced oxidative stress and apoptosis of HaCaT cells. R. Ahangari’s research proved that ellagic acid can protect cardiomyocytes from oxidative damage [52]. Hou et al. [53] reported the protecting effect of deoxyschisandrin and schisandrin b on HaCaT cells against UVB-induced damage. A study on the total flavonoids extract from Hedyotis diffusa found that flavonoids can protect cells by reducing the level of ROS in cells [54]. Therefore, in this case, the ellagic acid, lignans and flavonoids in the FB may positively contribute to the protection of HaCaT cells against the oxidative damage induced by H_2_O_2_.

### 2.6. Effect of FB and WE on ROS Level in HaCaT Cells Treated with H_2_O_2_

**Figure 6:**
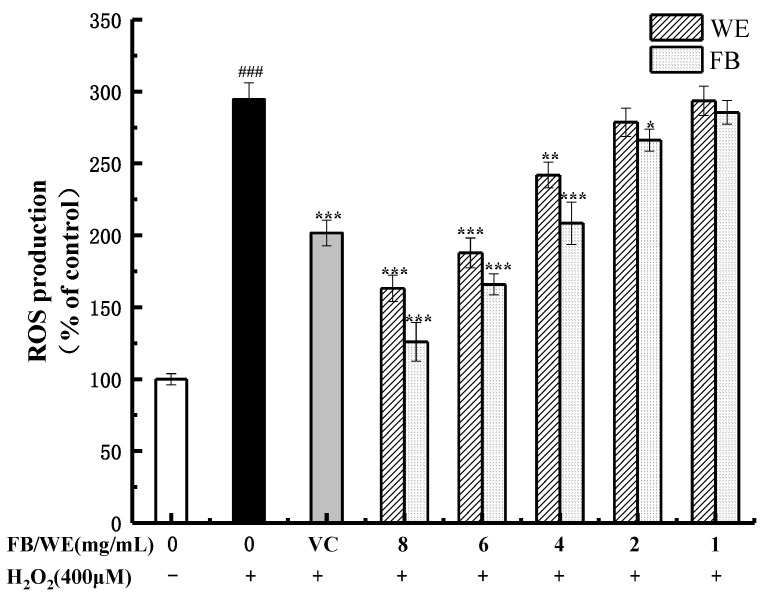
Effects of the WE and FB on H_2_O_2_-introduced ROS production in HaCaT cells. The cells were cultured with different concentrations (8, 6, 4, 2 and 1 mg/mL) of WE and FB for 24 h and then stimulated by 400 μM of H_2_O_2_ for 2 h. The ROS content was detected by DCFH-DA and examined by a fluorescence enzyme reader. In the picture: VC stands for Vitamin C, and the concentration is 2 μg/mL. Significance: ^###^ *p* < 0.001 vs. the control group. *** *p* < 0.001, ** *p* < 0.01 and * *p* < 0. 05 vs. the H_2_O_2_-treated group. Data shown as the mean ± S.D. (*n* = 5).

H_2_O_2_ can stimulate the production of ROS in HaCaT cells, resulting in an oxidative stress response and triggering the activation and apoptosis of various signaling pathways in HaCaT cells [55]. As shown in Figure 6, the fluorescence intensity of the H_2_O_2_-stimulated injury group (294.49% ± 11.54%, *p* < 0.001) was significantly higher than that of the control group, indicating that H_2_O_2_ has a great influence on the level of intracellular ROS. The addition of VC effectively inhibited the production of ROS (201.63% ± 8.88%, *p* < 0.001) and protected the cells. However, the levels of ROS in the HaCaT cells pretreated with different concentrations (1, 2, 4, 6 and 8 mg/mL) of WE and FB were lower than those pretreated with H_2_O_2_.

Especially when the concentrations of WE and FB were 8 mg/mL (WE: 163.15% ± 9.13%, *p* < 0.001; FB: 125.94% ± 13.46%, *p* < 0.001) or 6 mg/mL (WE: 187.85% ± 10.33%, *p* < 0.001, FB: 165.92% ± 7.4%, *p* < 0.001), the levels of intracellular ROS were lower than that of the VC group. The FB can effectively reduce the ROS levels in HaCaT cells induced by H_2_O_2_ so that FB has good cell protection against oxidative damage in the range of 4~8 mg/mL. A study showed that ellagic acid extracted from the leaves of Clerodendrum viscosum can inhibit the production of excessive ROS [56]. Similarly, Sepand’s [57] study also confirmed that ellagic acid can improve lipid peroxidation in renal tissue and protect the mitochondria from Gentamicin-induced mitochondrial damage by reducing the ROS content in the mitochondria.

## 3. Materials and Methods

### 3.1. Experimental Materials and Chemical Reagents

Pomegranate was sold as Tunisia soft-seed pomegranate, and *Schisandra chinensis* was sold as north *Schisandra chinensis* of Changbai Mountain by Tongrentang. In the pre-experiment, we found that the mixed extraction of *Schisandra chinensis* and pomegranate peel could increase the content of ellagic acid in the extract. Rinse pomegranates with water, cut them into thick slices with a knife and freeze them, then put frozen pomegranate peels into a vacuum freeze-drying machine (TF-FD-1, Tuoyuan, Shanghai, China) for freeze-drying. Wait until the pomegranates are crisp flakes, and then, the artificial skins are separated, and the pomegranates are collected and crushed into yellow powder by a shredder. Wash and dry *Schisandra chinensis*, crush into dark-red granules with a pulverizer, sift through 50 mesh, dry again, seal and refrigerate for use.

The chemical reagents used are as follows: NaOH, 2,2-diphenyl-1-picrylhydrazyl, H_2_SO_4_, NaNO_2_, Al(NO_3_)_3_, ABTS, ellagic acid standard, schisandrin B standard, analytical pure glucose powder, CCK-8 assay, DMEM, FBS, DPBS, DCFH-DA, anhydrous ethanol, methanol, chromic acid and other reagents, all purchased from MACKLIN (Shanghai, China), and the purity and manufacturer’s information can be seen in Supplementary Materials, Table S1. HaCaT cells were purchased from the Shanghai Cell Bank of the Chinese Academy of Sciences.

### 3.2. Preparation of FB

Accurately weigh pomegranate peel powder and *Schisandra chinensis* powder, each 2.5 g in a conical flask, add in 6 g of glucose and 100 mL of distilled water, put the sealed powder into a high-pressure sterilizer (DSX-280B, Shen’an, Shanghai, China) for 30 min, remove and place in an ultra-clean working table (BHC-1300B2, AIRTECH, Hsinchu County, Taiwan) and cool to room temperature, add in 0.5 mL each of *Lactobacillus Plantarum* and *Streptococcus Thermophilus*, put into a shaking table and culture at 37 °C for 24 h. A high-speed refrigerated centrifuge (TGL-16M, Luxiangyi, Shanghai, China) was used to treat the FB to get the aimed fermentation liquid.

### 3.3. Determination of Ellagic Acid in the FB

In this study, ellagic acid was measured by an ultraviolet spectrophotometer (ALPHA-1860, Puyuan, Shanghai, China). The content of ellagic acid was determined by referring to Liu’s experimental method and making improvements [58].

### 3.4. Determination of Antioxidant Activity

In this study, the DPPH radical scavenging rate was measured by an ultraviolet spectrophotometer [59]. In this experiment, DPPH was a major antioxidant index. At the same time, other antioxidant indices, such as ABTS^+^ [60], OH^−^ [61] and O_2_^−^ [62], were determined to confirm the strength of the antioxidant activity.

### 3.5. Characterization of Ellagic Acid by HPLC

The instrument used in this experiment was the Agilent 1260 high-performance liquid chromatography system (Agilent Technologies Inc, Walter Bloom Germany). Chromatographic conditions: C18 column; mobile phase: acetonitrile (a): 0.1% phosphoric acid and (b) (18:82), flow rate: 1 mL/min, column temperature: 25 °C, detection wavelength: 254 nm and injection volume: 20 μL. Column specifications: 5-μm Eclipse Plus C18 column (Agilent, Santa Clara, CA, USA); The HPLC separation type: gradient mode; 0.025 mg/mL of ellagic acid standard solution was prepared with the HPLC grade methanol. The WE and FB were made into freeze-dried powder, and then, the mixture was made into 10 mg/mL solution.

### 3.6. Effects of FB, WE and H_2_O_2_ on the Activity of HaCaT Cells

The activity of the HaCaT cells was determined by the CCK-8 assay. The HaCaT cells were cultured in DMEM medium containing 10% fetal bovine serum. The cells were seeded in 96-well plates according to the density of 8000 per well. Then, the plates were placed into the carbon dioxide incubator at 37 °C and 5% CO_2_, and the CCK-8 assay was added [63]. After incubation for 4 h, the optical density (OD) value was measured at the wavelength of 450 nm by the enzyme reader, which was expressed as the percentage of HaCaT cell activity in the control group [64].

To determine the nominal concentration of the H_2_O_2_-induced oxidative damage model in vitro, the HaCaT cells were treated with a nominal concentration of H_2_O_2_ (50, 100, 200, 300, 400, 500, 600, 700 and 800 μM) for 2 h [65], and then, the cell survival rate was detected by the CCK-8 assay.

The WE and FB with the same concentrations were filtered by a 220-nm microporous membrane, and the two were prepared into a mixed medium with a concentration of 10, 8, 6, 4, 2, 1, 0.5 and 0.1 mg/mL by using a complete medium. To explore the oxidative protection, HaCaT cells were pretreated with a mixed medium for 24 h before oxidative damage [66] and then exposed to the optimal concentration of hydrogen peroxide After 2 h, the CCK-8 assay was used to determine the cell activity.

### 3.7. Effects of FB and WE on the ROS Level in HaCaT Cells Treated with H_2_O_2_

The level of ROS in the HaCaT cells was detected by a fluorescent probe DCFH-DA. The cells were seeded according to the density of 8000 per well. After 24 h of culture in the mixed medium of 8, 6, 4 and 2 mg/mL, the cells were stimulated with 400 μM H_2_O_2_ for 2 h, and then, the DCFH-DA fluorescent probe was added [67]. After 20 min of incubation in the incubator, the cells were washed with DMEM medium, and then, the fluorescence intensity was detected in the fluorescence enzyme reader. The emission wavelength was 488 nm, and the excitation wavelength was 525 nm [68].

### 3.8. Experimental Design and Statistical Analysis

Design-Expert software 7.0 was used for the experimental design. The accuracy of the polynomial model equation was checked by the F-test and *p*-value. Statistically significant: *p* < 0.05. All measurements were taken three times. Origin 2018 (Softonic International, Barcelona, Spain) data processing software was used for data processing and analysis.

## 4. Conclusions

In the paper, the RSM coupled with Box-Behnken design was applied successfully to optimize the fermentation process of pomegranate peels and *Schisandra chinensis*. In this work, three factors were evaluated. The optimized condition was obtained and verified with the experimental value. The lactobacillus fermentation more effectively released ellagic acid compared to the water extraction, which was further verified by the application of HPLC to quantify the ellagic acid. The FB showed stronger free radical scavenging abilities against the ABTS^+^, DPPH, OH^−^ and O_2_^−^ free radicals. Under the concentration without cytotoxicity, both the FB and WE had protective effects on the H_2_O_2_-injured HaCaT cells, and the FB showed a stronger cell-protective effect. This paper provided the data support for the preparation technology and bioactivity for the FB of PP&SC. The efficient preparation process, outstanding antioxidant ability and protective effect against H_2_O_2_-induced oxidative damage in HaCaT Cells could promote the application of the FB of PP&SC as functional raw materials of cosmetics.

## Figures and Tables

**Table 1:** Response surface design and experimental results.

Serial Number	A/g	B/h	C/°C	Ellagic Acid Content (mg/g)
1	3	36	37	95.25
2	6	48	37	84.29
3	3	24	35	83.04
4	3	48	35	79.86
5	3	36	37	96.41
6	0	36	39	82.29
7	0	48	37	83.94
8	3	36	37	95.48
9	6	36	39	83.53
10	0	36	35	80.71
11	0	24	37	86.31
12	6	36	35	84.73
13	3	25	39	86.75
14	3	48	39	82.62
15	3	36	37	97.80
16	3	36	37	97.22
17	6	24	37	90.33

A = Glucose dosage (g/100 mL), B = fermentation time (h) and C = fermentation temperature (°C).

**Table 2:** Response surface quadratic model ANOVA and regression coefficient estimation.

Parameter	Sum of Squares	df	Mean Squares	F-Value	*p*-Value
Model	625.19	9	69.47	50.22	<0.0001 ***
A	11.59	1	11.59	8.38	0.0232 *
B	30.89	1	30.89	22.33	0.0021 **
C	5.87	1	5.87	4.24	0.0483 *
AB	3.37	1	3.37	2.43	0.0027 **
AC	1.93	1	1,93	1.40	0.0258 *
BC	0.23	1	0.23	0.16	0.0184 *
A^2^	115.32	1	115.32	83.37	<0.0001 ***
B^2^	104.46	1	104.46	75.52	<0.0001 ***
C^2^	295.93	1	295.93	213.94	<0.0001 ***
Residual	9.68	7	1.38		
Lack of Fit	4.89	3	1.63	1.36	0.3795
Pure Error	4.80	4	1.20		
Cor total	634.87	16			
R^2^	0.9820				

A = Glucose dosage (g/100 mL), B = fermentation time (h), C = fermentation temperature (°C) and R^2^ = Coefficients of determination. Significance * *p* < 0.05, ** *p* < 0.01 and *** *p* < 0.001.

## Data Availability

Not applicable.

## Supplementary Materials

The following are available online. Table S1: Reagent information.

## Sample Availability

Samples of the fermentation broth and water extracts are available from the authors.
