# Supplementary material for: Optimization of Fermentation Process of Pomegranate Peel and Schisandra Chinensis and the Biological Activities of Fermentation Broth: Antioxidant Activity and Protective Effect Against H2O2-induced Oxidative Damage in HaCaT Cells
Source: Molecules. 2021 Jun 5;26(11):3432. doi: 10.3390/molecules26113432 (PMC8201020; doi:10.3390/molecules26113432)
Supplement: Supplementary file 1 [file molecules-26-03432-s001.zip › molecules-1212665-supplementary.pdf]

molecules

Table S1. Reagent information in the article.

Name	Purity Grade	Manufacturer Information
NaOH	≥96%(AR)	Greagent
2,2-diphenyl-1-picrylhydrazyl	/	CATO
ABTS	/	Beyotime
Ellagic acid standard	≥ 98%(HPLC)	STANDARD
Schisandrin B standard	≥ 98%(HPLC)	STANDARD
Analytical pure glucose powder	≥ 98%	Alfa
CCK-8 assay	/	Beyotime
DCFH-DA	/	Beyotime
H_2O_2	31%(G2)	Adamas
HCl	37%(G1)	Adamas
Salicylic acid	99%(RG)	Adamas
$FeSO_4 \cdot 7H_2O$	≥99.0%(AR)	Greagent
$K_2S_2O_8$	≥99.5%(AR)	Greagent
Anhydrous ethanol	≥99.7%(AR)	Greagent
Pyrogallol	98%+(RG)	Adamas
$Na_2HPO_4 \cdot 12H_2O$	≥99%(AR)	Greagent
NaH_2PO_4	≥99.0%(AR)	Greagent
DMEM	/	Gibco
FBS	/	Gibco
DPBS	/	Gibco
DMSO	/	Solarbio
Broth medium	/	HaiBo
